# MRI Characteristics of Split Cord Malformation

**DOI:** 10.7759/cureus.18328

**Published:** 2021-09-27

**Authors:** Ahmad Jiblawi, Hani Chanbour, Azzam Tayba, Haissam Khayat, Khaled Jiblawi

**Affiliations:** 1 Radiology, American University of Beirut Medical Center, Beirut, LBN; 2 Faculty of Medicine, Lebanese University, Beirut, LBN; 3 Radiology, Beirut Arab University, Beirut, LBN

**Keywords:** spinal canal, malformation, hemicord, spinal dysraphism, split cord malformation

## Abstract

Split cord malformation (SCM), a subtype of spinal dysraphism, represents a rare entity of congenital malformation in the spine that occurs rarely in adults. SCM commonly presents with back pain. MRI is the gold standard for diagnosis. We report a case of a previously healthy 25-year-old woman who presented with lower back pain for a duration of 6 months. MRI confirmed a diagnosis of type I SCM. This case highlights the neuroimaging characteristics of SCM, although it might remain asymptomatic for many years. Surgical intervention might not be indicated in case of absent clinical complications.

## Introduction

Split cord malformation (SCM) is a rare dysraphic lesion of the spinal cord. It consists of dividing the spinal cord into two symmetrical or asymmetrical hemicords [1], produced by the presence of an osseous or fibrocartilaginous septum in the central portion of the spinal cord creating a complete or incomplete sagittal division [2]. Each hemicord contains the central canal, an anterior horn, and a posterior horn and is coated with its own layer of pia matter [1,3]. The origin of the defect is an abnormal development of the notochord between the 15th and 18th day of pregnancy [3,4].

The condition was first reported in 1837 [5,6]. It can be prenatally diagnosed during the 14th week of gestation [4]. It is more common in women and most frequently found in the lumbar region [1]. SCM is classified into type I and type II; the first symptomatic entity is characterized by the presence of a bony or fibrous spur with separate dural sacs and arachnoidian space surrounding each bead, in contrast, the latter is most commonly asymptomatic or rarely symptomatic with no evidence of the presence of the bone spur [1,7].

SCM might occur isolated, which confers a better prognosis [3] or it might be accompanied by other spinal dysraphisms, such as meningocele, myelomeningocele, neuroenteric cysts, or spinal lipoma, as well as vertebral anomalies including scoliosis, hemivertebrae, and butterfly vertebrae [1,3,7].

The literature shows numerous cases of SCM diagnosed in the prenatal period by means of ultrasound and confirmed by MRI [4,8,9]. Majority of patients are under the age of 7; however, the oldest reported case of SCM was for a 78-year-old lady [3]. Currently, there are 146 unique adult cases of SCM reported [10]. We herein describe a case of a 25 years old female patient presenting with SCM.

## Case presentation

We report a case of a 25-year-old lady who presented for a lower back pain of 6 months duration. Physical exam was unremarkable except for the lower back pain. There were no signs of radiculopathy, lower extremity weakness, or bowel/bladder dysfunction. Neurologic exam was normal at presentation.

**Figure 1:**
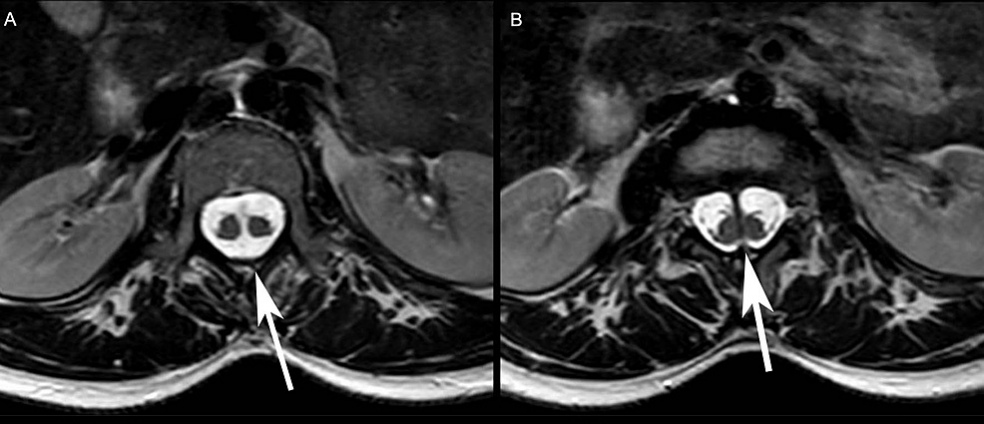
A: T2-weighted image, axial plane, level of L2, shows a single dural sac containing both hemicords with appearance of the chondro-osseous ligament central in anteroposterior orientation. B: T2-weighted image, axial plane, level of L2–L3, shows two hemicords separated by the chondro-osseous ligament, each in a separate dural sac.

**Figure 2:**
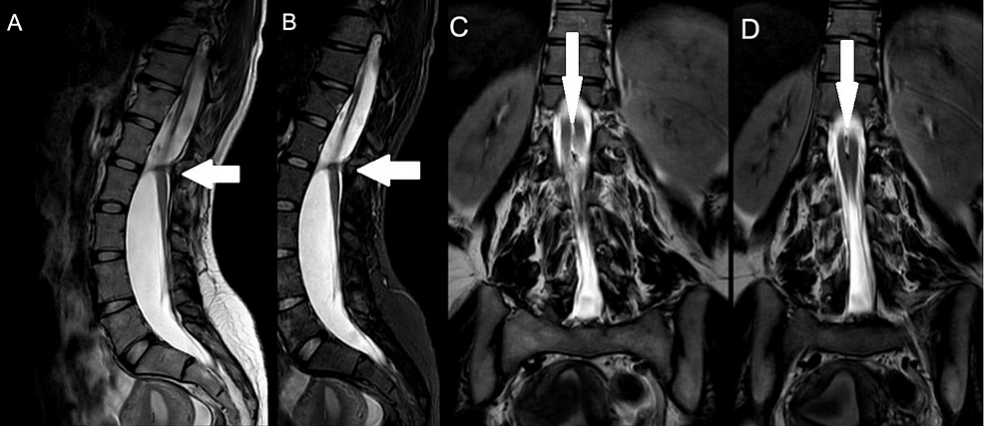
A: T2-weighted image, sagittal plane, shows the presence of a chondro-osseous ligament at the level of L1–L2. B: Short T1 inversion recovery image, sagittal plane, shows the presence of a chondro-osseous ligament at the level of L1–L2. C: T2-weighted image, coronal plane, shows the sagittal division of the spinal cord into two hemicords. D: T2-weighted image, coronal plane, shows the bifid cord joining distally.

MRI of the lumbar spine was performed on a 3T Ingenia unit (Philips, Netherlands) in the following planes and sequences: 1) sagittal T1 turbo spin-echo (TSE), T2 TSE, short tau inversion recovery fat suppression (STIR), 2) axial T1 TSE, T1 fat-saturated, T2 TSE, 3) coronal T2 TSE. There was evidence of SCM (type I) at L1-L2 level (Figures 1,2), with a genuine chondro-osseous ligament central in anteroposterior orientation, dividing the canal and cord at this level, with the bifid cord joined together adequately proximally and distally, remaining of normal signal and showing no evidence of hydrosyringomyelia. There’s no intra- or extramedullary masses or lipoma. The spinal canal is congenitally wide from L1 and downwards without any associated myelomeningocele. Additionally, the vertebral bodies were of normal outlines and signal, without accompanying vertebral collapse or fractures. Intact posterior elements and vertebro-pedicular junctions were also noted.

This patient had an isolated SCM without any other associated spinal cord dysraphisms or vertebral column malformation.

## Discussion

The classification of SCMs was simplified by Pang in 1992 by dividing it into type I and type II. The factors used to differentiate between those two types of SCMs are the presence of one or two dural tubes and whether the dividing median septum is rigid or non-rigid. Type I SCM consists of two hemicords, each contained in a distinct dural tube divided by a rigid osseocartilagenous median septum. Type II SCM implies housing of two hemicords in a single dural tube separated by a non-rigid fibrous median septum. Owing to the rarity of this condition, inconsistencies arose in the literature mainly in three recurrent areas: the nomenclature; embryological origin; and clinical significance. As a result, terms like diastematomyelia, diplomyelia, and dimyelia were used interchangeably and are currently substituted by the term SCM. Concerning the origin, the wide accepted theory proposes that the origin of all SCMs originated from one basic ontogenic error occurring around the time of closure of the primitive neurenteric canal. A formation of an accessory neurenteric canal which is considered as an abnormal fistula causes regional splitting of the notochord and the overlying neural plate [7-10]. The location of this abnormality is highly variable, having the lumbar region being the most common (51.9%) and the cervical and sacral regions being the rarest (0.9%). As for its clinical significance, the most commonly reported symptom was neck/back pain (68.5%), followed by radiculopathy/paresthesia (51.8%) and lower extremity weakness (50.9%). It is rare for this condition to cause presence with impotence (1.8%) and it was diagnosed since birth in only two cases [10]. Our patient can be considered as a typical example of split cord malformation, she is a woman (74.7%), aged 27 corresponding to the mean age of the literature review (26.8 ± 2.9), presenting with lower back pain without radiculopathy for a duration of 6 months (10.3 ± 3.9). Our patient didn’t have any associated spina bifida, cutaneous lesions, spinal deformity, or lipoma which are known to be concomitant with split cord malformation. However, according to the literature, the most common associated conditions were: tethered cord (59.8%) and hypertrichosis (44%). Plain X-ray and CT scan exams were not performed in our case. MRI is the gold standard modality for identification and visualization of SCMs and neural elements, respectively [10]. In our patient, MRI revealed split cord malformation at level of L1-L2 due to a chondro-osseous ligament central in anteroposterior orientation, dividing the canal and cord at this level. This is consistent with a type I SCM, where there is a distinct dural sac surrounding each hemicord (Figure 1B). Management is usually conservative. However, surgical options could offer better outcomes in cases with other associated abnormalities, in terms of neurologic function and pain improvement [10].

## Conclusions

Split cord malformations represent a rare entity of spinal dysraphisms. The condition can be prenatally diagnosed, which offer early recognition thus better treatment outcome. Type 1 and type 2 SCMs are differentiated by the presence of two dural sacs surrounding each hemicord in type 1, compared to one dural sac in type 2. This case report represents an additional example of type I SCM presenting in adulthood, and as far as our knowledge, it is the first case to be reported in Lebanon.
